# Supplementary material for: Small circRNAs with self-cleaving ribozymes are highly expressed in diverse metazoan transcriptomes
Source: Nucleic Acids Res. 2020 Mar 21;48(9):5054–64. doi: 10.1093/nar/gkaa187 (PMC7229834; doi:10.1093/nar/gkaa187)
Supplement: gkaa187_Supplemental_File [file gkaa187_supplemental_file.pdf]

Supplementary Table 1. List of the adjacent and divergent oligos used for the amplification of genomic retrozymes and retrozyme circRNAs.

Oligo name	Oligo sequence
108D_Coral_Rtz	TACCACATKTGTGGGAMRYTYGRGGT
108R_Coral_Rtz	CTMAGYCMTGCCAGAGTGCTCRAACYA
109D_HH_Mytil_Rtz	TGGCGTATATAYAAAATTTAGTCCTG
109R_HH_Mytil_Rtz	GACGCGCGTTTCGTCTACAAAAGAC
110D_Mytil_Rtz	GACATGAAYTRTCATTGATATKGTTATA
110R_Mytil_Rtz	GCTGACTACTDGGCTKGTGATACCYTYGG
144D_Amex_Rtz350U_BAMHI	TATGTGGGGATCCTTCTTCTCTGTGAAAAGAAC
144R_Amex_Rtz350U_ECORI	CATATCAGAATTCGTCCTGCCCAGGGGCAGTC
144Rb_Amex_ShortRtz350U	TTAGTCTTTGTCCTGCCCAGGGGC
145D_Amex_Rtz330G_BAMHI	GTTAGATGGATCCGAAACCTTCCTCCCATC
145R_Amex_Rtz330G_ECORI	CCACTGGGAATTCCTCTGGGCCACCTTCC
149D_DimerAmex330	TTTAGATTCATTCGAAACCTTCCTCCCATCAC
149R_DimerAmex330	CCACTGGCAATTGCTCTGGGCCACCTTCCAC
150D_DimerAmex350	GGGGATATTTCTTCTCTGTGAAAAGAACAG
150R_DimerAmex350	ACATATCAGTCTTTGTCCTGCCCAGGGGCAGTC

Supplementary Table 2. Constructs used to synthesize the retrozyme RNAs, markers and probes used in this work. Polylinker sequences are shown in red, whereas retrozyme sequences are shown in black. The sites of HHR self-cleavage for each retrozyme sequence are marked with a blue vertical line.

<table>
<tr><th>Construct</th><th>Sequence</th></tr>
<tr><td>Rtz Genom coral Amil224</td><td>T7_promoter_GGGCGATTGGAGCTCCACCGCGGTGGCG
GCCGCTCTAGAACTATACCACATGTGTGGGACACTCGAGG
TAGCCTTATAGGCTTACCTTCATCCTAGCTATCAGCACTG
CACTGATGAGGCCCAGAAGGCCGAAACAGTACTGTC|TGC
AGTTAGATATAGCTCTTTGGTGTAAAAACCAAATCGAGCA
CTGTACTGGGATGAGTCATTGCCGATAGCAATTGCGCACG
CTCACTAGTTCTCTGGTTCGAGCACTCTGGCATGACTTAG
TAGTGGATCCCCCGGGCTGCAGGAATT</td></tr>
<tr><td>Rtz Genom mussel Mg353</td><td>T7_promoter_GGGCGATTGGAGCTCCACCGCGGTGGCG
GCCGCTCTAGAACTAGACATGAATTGTCATTGATATGGTT
ATATTTATAAATTTACTGTTTACAAATTTTTGAATTTTTT
GAAATACTAAGGCTTTTCTACCTCATGCATAGATTACCTT
AGCTGTATTTGGCAAAACGTTTAGGAATTTTGGTTTTCAA
TGCTCTTCAACTTCGTACTTTATTTGGCCTTTTTAACTTT
TTTGGATTCGAGCGTCACTGATGAGTCTTTTGTAGACGAA
ACGCGCGTC|TGGCGTATTTACTAAATTTGGTCCTGATAT
CTATGATGAGTTTATTTGCAACCACTGGGTCGATGCCACT
GCTGGTGGAGATTTATTTCTCCGAGGGTATCACCAGCCCA
GTAGTCAGCTAGTGGATCCCCCGGGCTGCAGGAATT</td></tr>
<tr><td>Rtz Genom axolotl Ax353</td><td>T7_promoter_GGGCGATTGGAGCTCCACCGCGGTGGCG
GCCGCTCTAGAACTAGTGGATCCTTCTTCTCTGTGAAAAG
AACAGTGAGCTAAAGACTTGGTTGAGACTCTCCCAGGGGC
CATCCACCCAAGAACAAGTCAATGGGCTGTGTTTGTGCTG
GTAGAAAGTTTCCCAGAACCCCCCTGTTTTGCATACCAAG
TAACTTTGGGTCTCACCCTAAGTTACCAGGGGACTCCAGA
CGTGGACCCCTTGCTCACTGCTCTTAGAGAGTCACAGCTT
CACCTCACTGATGATGCCCAACTAGGCTGAAACACGTGTA
|TGGGGTTGCTGTGGTACTCTTGTTCAGAGGAGAATTGCC
TAGCATTTCGGTGCTGGACTGCCCCTGGGCAGGACGAATT</td></tr>
<tr><td>Rtz Genom axolotl Ax330</td><td>T7_promoter_GGGCGATTGGAGCTCCACCGCGGTGGCG
GCCGCTCTAGAACTAGTGGATCCGAAACCTTCCTCCCATC
ACTTTTGTGGTCAGTTTGTTTCTCCCTCTGAGAGAGGGTA
TGCCCAGACGTGGGTCCCTTGCTCGCTGCGCCACAGGGAA
CCAGGCTGCACCTCACTGAGGAGGTCCAAAAAGGCCGAAA
CATGTC|TGGGGTTGCTTGGTTTTCTGTGCAGAGGGGACC
TGGCCTAGCAGTTCGGGCTGGGCTGCTACCTTCGGTGGCG
GCAGCTGCTGACTGATTTGCACATGGCTGGTCCCCTTTTG
CAATGGCGCAGACGGGCTAAAAAACGGTGGTGTGGAAGGT
GGCCCAGAGGAATT</td></tr>
</table>

Legends for Supplementary Figures.

Supplementary Figure S1

A selected group of the major type I HHR families detected in the genome of the coral *Acropora millepora*. Family 1 motifs (boxed inset) correspond to the canonical type I HHR present in retrozymes (~200 bp repeats), whereas all the other families of minimal HHRs are related to autonomous retrotransposons, such as PLEs or Terminons. The consensus sequence (70% homology) of the aligned HHRs for each family is shown. Only those sequences allowing a perfect helix topology and showing the conserved catalytic center (CUGANGA / GAAAY / RUH) were used for the HHR alignments. Nucleotides in red correspond to the conserved non-canonical (47) loop-loop interactions of type I HHRs.

Supplementary Figure S2

(A) RNA extracts from two different samples of the coral *Acropora millepora* (Am_RNAs_1 and Am_RNAs_2, 10 μg each) were run in a native PAGE (left), and the regions corresponding to the retrozyme RNAs (200-300 bp, see Figure 2B) were cut from the gel (center) and purified. The resulting purified RNAs from each extract were amplified by RT-PCR (right gel, amplification bands of 200-250 bp) using an adjacent and divergent pair of oligos (see Material and Methods for details). Negative amplification controls correspond to PCRs of the purified RNAs in the absence of an RT reaction (Ctrl (RT-)), RT-PCR in the absence of any RNA (Ctrl RNA-) and PCR with neither RT nor RNA samples (PCR-). (B) Sequence alignment of selected retrozyme sequences from 12 different species of stony corals. The first position of each sequence corresponds to the self-cleavage site of the retrozyme HHR. The highest sequence homology occurs among either the ribozyme motifs or the central region of the retrozymes (positions ~100-150 nt). (C) Minimum free energy secondary structure predictions of circRNAs derived from genomic retrozymes sequences from diverse stony corals. These sequences were detected either in this work or hypothesized in our previous publication (e. g. *A. digitifera* and *P. strigosa*) (26). Sequences corresponding to the hammerhead ribozyme are shown in purple.

Supplementary Figure S3

Minimum free energy secondary structure of a retrozyme circRNA sequence predicted from a genomic retrozyme copy of the anemone *Nematostella vectensis*. The sequence corresponding to the hammerhead ribozyme is shown in purple.

Supplementary Figure S4

Consensus sequences of two type I HHRs detected in the retrozymes of the *Mytilus galloprovincialis* genome (HHR A and HHR B consensus results from 2,880 and 1,681 motifs respectively). Both motifs drawn on the left correspond to essentially the same sequence, except for changes in loops 1 and 2 (nucleotides in red), and the upper stem of the helix I (nucleotides in grey). The putative nucleotides involved in tertiary interactions between loops 1 and 2 in most type I HHRs are circled. A feasible tertiary interaction between loops 1 and 2 of HHR A, which is conserved in most type I HHRs, is indicated with a dotted line. (R: A or G, Y: C or U, W: A or U, K: G or U).

Supplementary Figure S5

(A) Example of an RNA extract (~100 μg) from a mussel gonad run in a preparative native PAGE. The region corresponding to the retrozyme RNAs (300-400 bp) was cut and purified (left). The resulting purified RNAs from two different male specimens were amplified by RT-PCR (observed bands around 400 bp for RT-PCR1 and RT-PCR2 in the gel at the right) using a pair of adjacent and divergent oligos (see Material and Methods for details). Negative amplification controls correspond to PCRs of the purified RNAs in the absence of an RT reaction (PCR (RT-)) and PCR with neither RT nor RNA samples (PCR(-)). (B) Sequence alignment of the cloned circRNAs from the two RT-PCR amplifications. The obtained sequences were reordered, and the first position of each sequence in the alignment correspond to the site of HHR self-cleavage. The consensus sequence is shown at the bottom of the alignment.

Supplementary Figure S6

Minimum free energy secondary structure of some circRNAs encoded by putative retrozymes in the genomes of invertebrates from different phyla, such as (A) rotifers, (B) trematodes, (C) insects, (D) annelids, (E) planarians or (F) crustaceans. The sequence corresponding to the hammerhead ribozyme is shown in purple.

Supplementary Figure S7

(A) Sequence alignment of two pairs of retrozyme sequences from the axolotl (*Ambystoma mexicanum*) genome. Each pair corresponds to the Rtzm330 (G327 and G331) and Rtzm350 (U348 and U351) families. The consensus sequence is shown at the bottom. (B) RNA extracts from three different tissues of *A. mexicanum* (muscle, liver and gonad, 10 μg each) were run in a native PAGE (left), and the regions corresponding to the retrozyme RNAs (200-300 bp) were cut from the gel (center) and purified. The resulting RNAs from each tissue were amplified by RT-PCR (right gel, amplification bands of ~330 bp) using an adjacent and divergent pair of oligos (see Material and Methods for details). Negative amplification controls correspond to

PCRs of the purified RNAs in the absence of either RNA (Ctrl(RNA-), RT reaction for each RNA sample (Ctrl (RT-)), and PCR with neither RT nor RNA samples (Ctrl(PCR-)). (C) Consensus sequences (70% homology) of four families of minimal HHRs characteristic of PLE and Terminon retrotransposons detected in the axolotl genome.

Supplementary Figure S8

(A) Denaturing PAGE of an *in vitro* transcription of the dimeric construct DRtz_Mg353 from mussel (see scheme of the construct at the bottom of the panel). The band corresponding to the linear monomeric RNA Mg353 nt resulting from double self-cleavage was excised and purified. (B) Denaturing gel showing a RNA circularization experiment of the retrozyme monomer Mg353 in the absence (lane 1) and in the presence (lane 2) of RtcB ligase. A detail of the lane 2 of the gel after silver staining is shown on the right. (C) Native PAGE with ~200 ng (~50 ng in the case of the circRNA of mussel) of purified circular and linear RNAs of axolotl (Ax353) and mussel (Mg353) monomeric retrozymes. Despite their identical sizes, the Ax353 forms migrate slightly faster than the Mg353 ones, possibly because of a different degree of tertiary structure. Purified linRNA Ax740 molecule, corresponding to the full and uncleaved dimeric linear RNA from the axolotl construct DRtz_Ax353 (see Figure 5A), was included as a reference. (D) Autoradiography of self-ligation experiments performed with a radiolabelled monomer RNA Mg353. Experiments were performed in the absence of Mg^{2+} (lane 1), with 10 mM Mg^{2+} (lane 2) and with 50 mM Mg^{2+} (lane 3). Dimeric, circular and linear RNA molecules are indicated..

Supplementary Figure S9

A tentative model for the replication cycle of metazoan (non-LTR) and plant (LTR) retrozymes. Either non-LTR retrozymes carrying type I HHRs described in metazoans or LTR retrozymes carrying type III HHRs described in plants could follow a similar propagation pathway through circRNAs as retrotransposition intermediates. These circRNA intermediates would be reverse transcribed as oligomeric cDNA repeats, and integrated at a new genomic locus by the machinery of autonomous retrotransposons present in the genome of the host.

Type I HHRs in the genome of the coral *Acropora millepora*

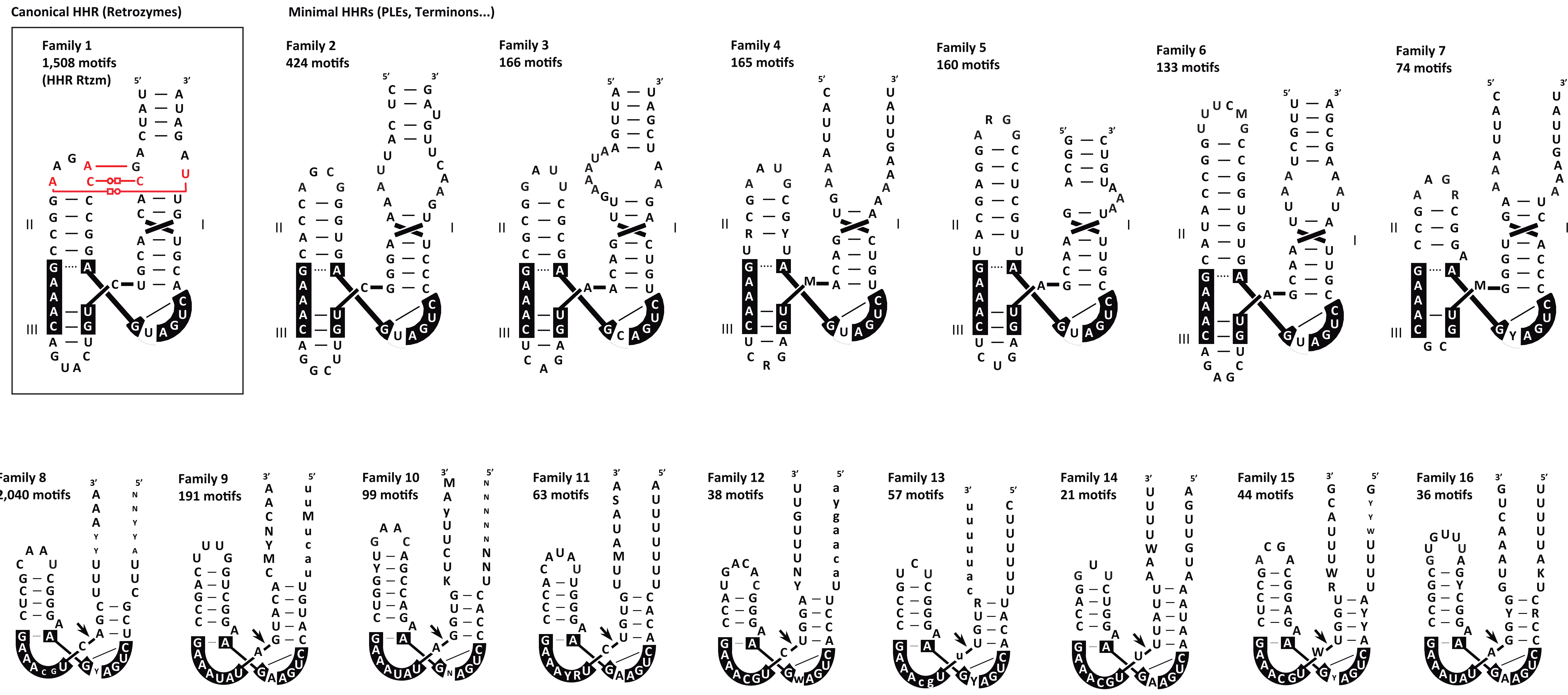

Supplementary Figure S1

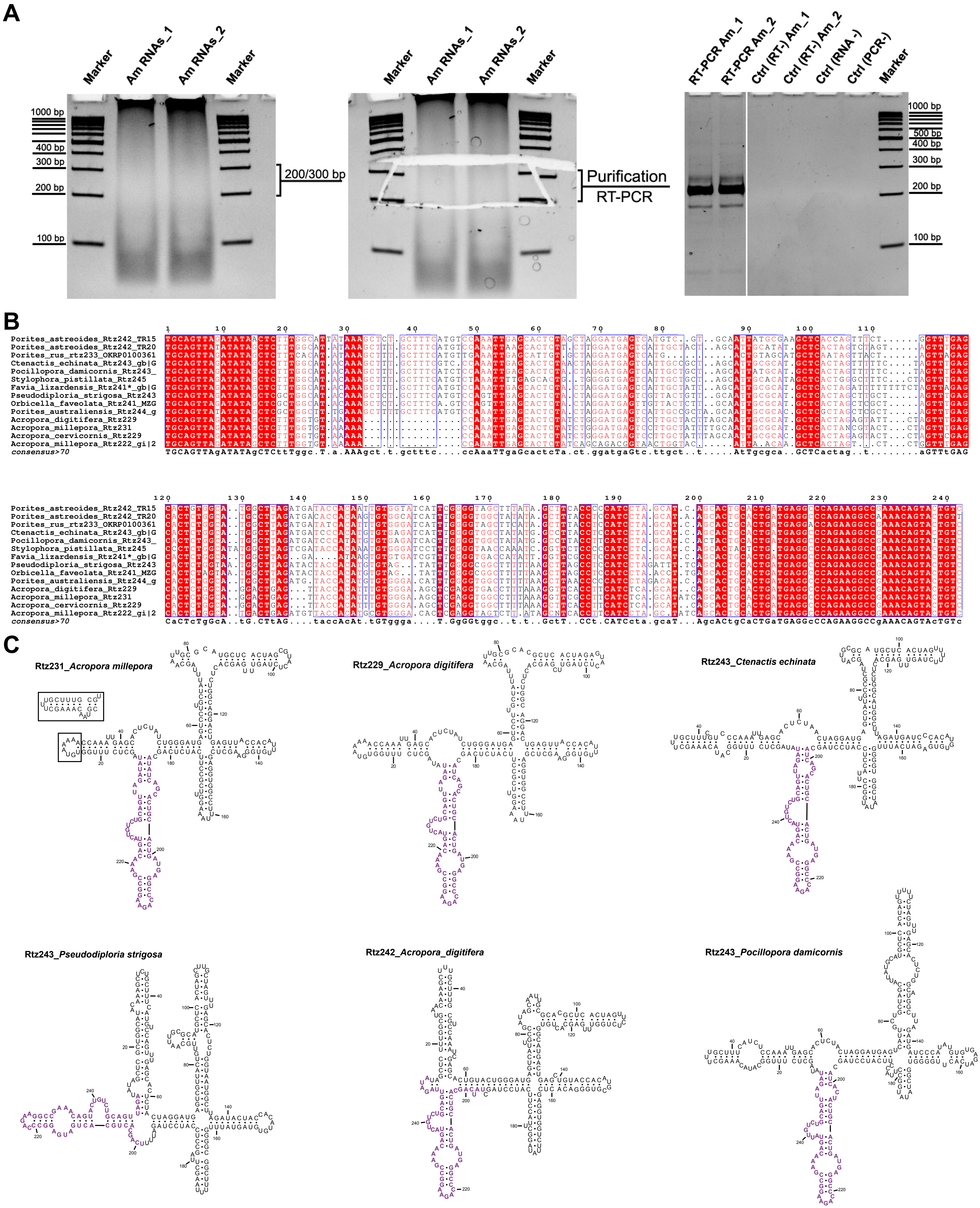

Supplementary Figure S2

Rtz354_*Nematostella vectensis*

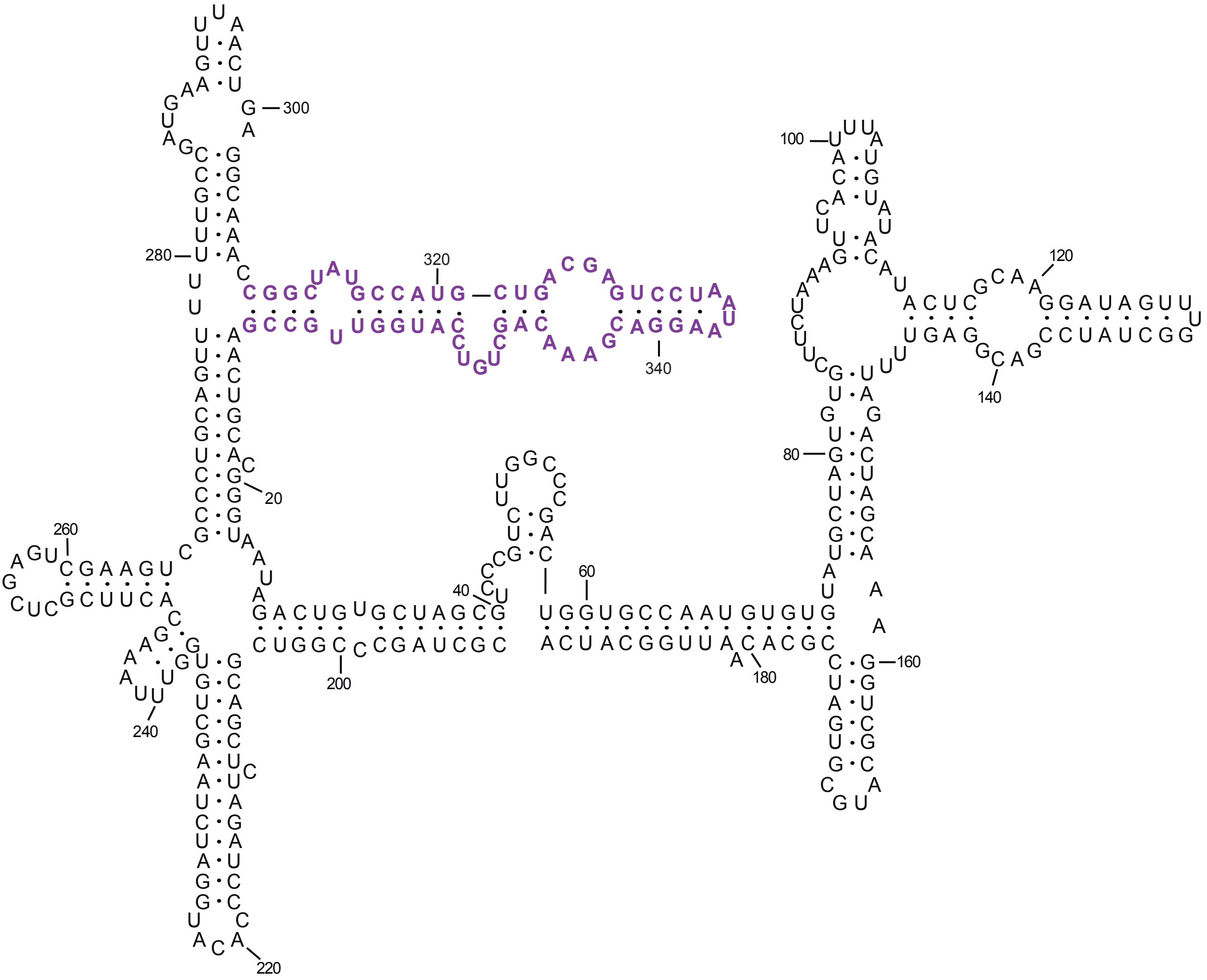

Supplementary Figure S3

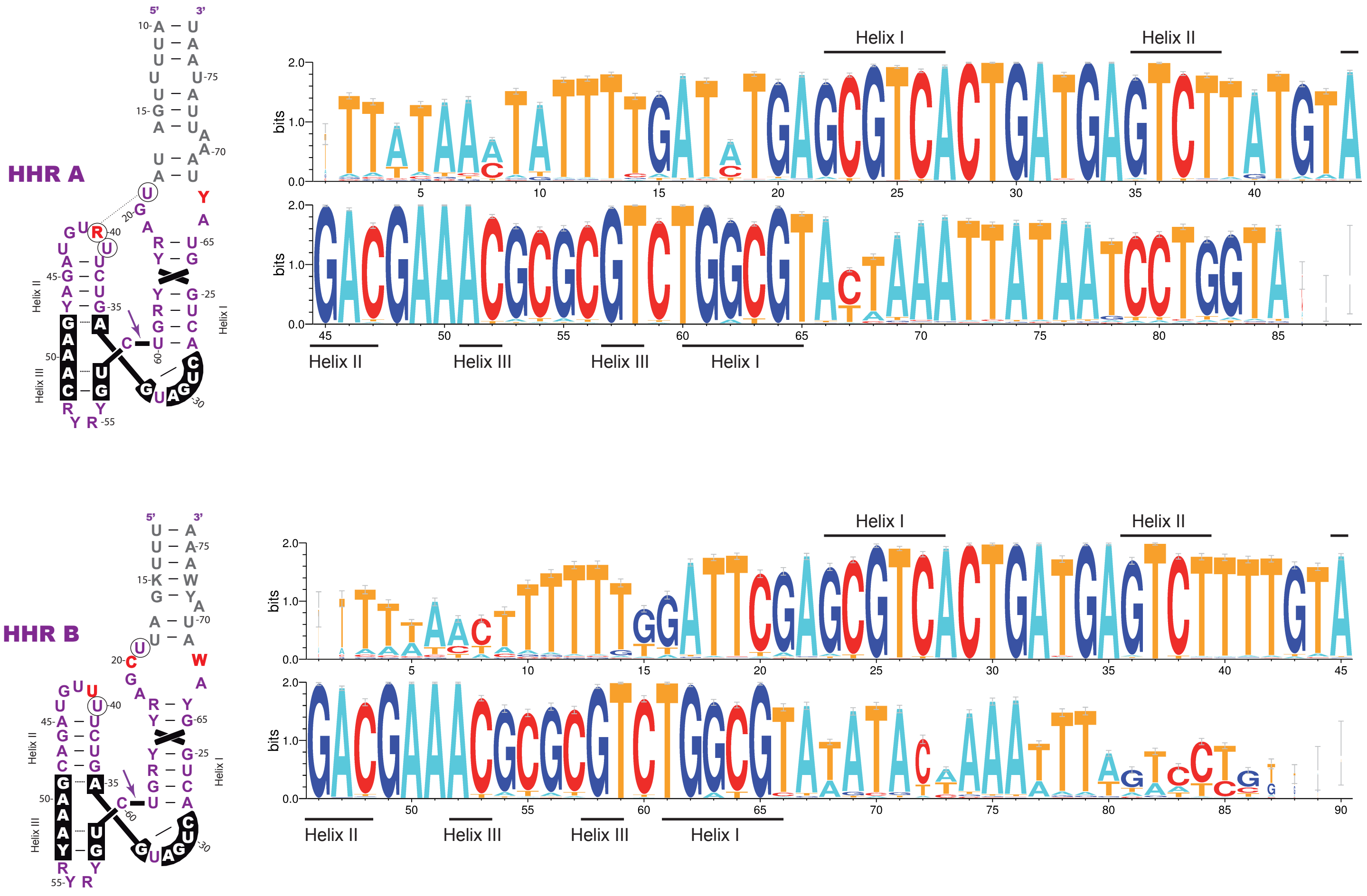

Supplementary Figure S4

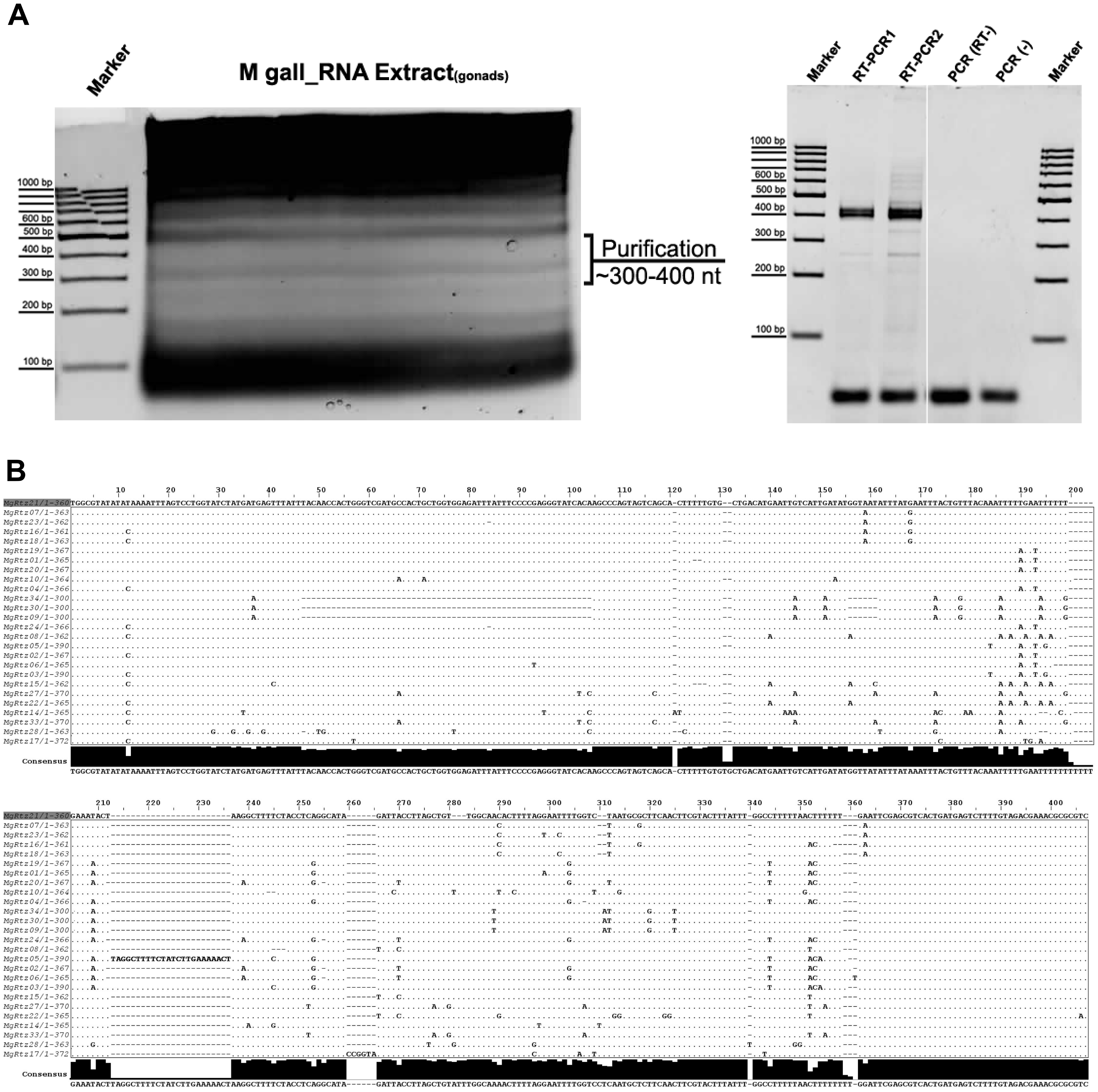

Supplementary Figure S5

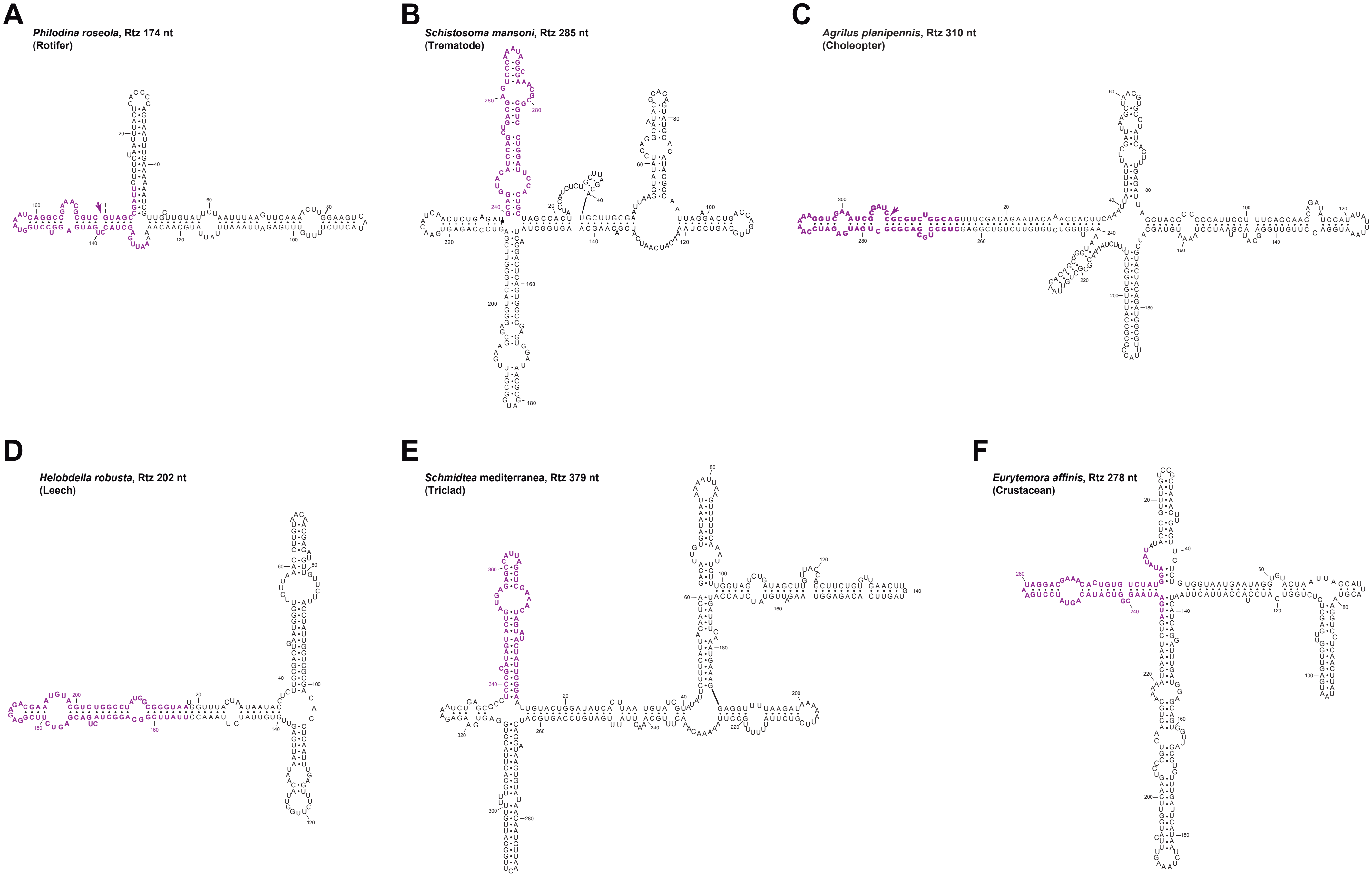

Supplementary Figure S6

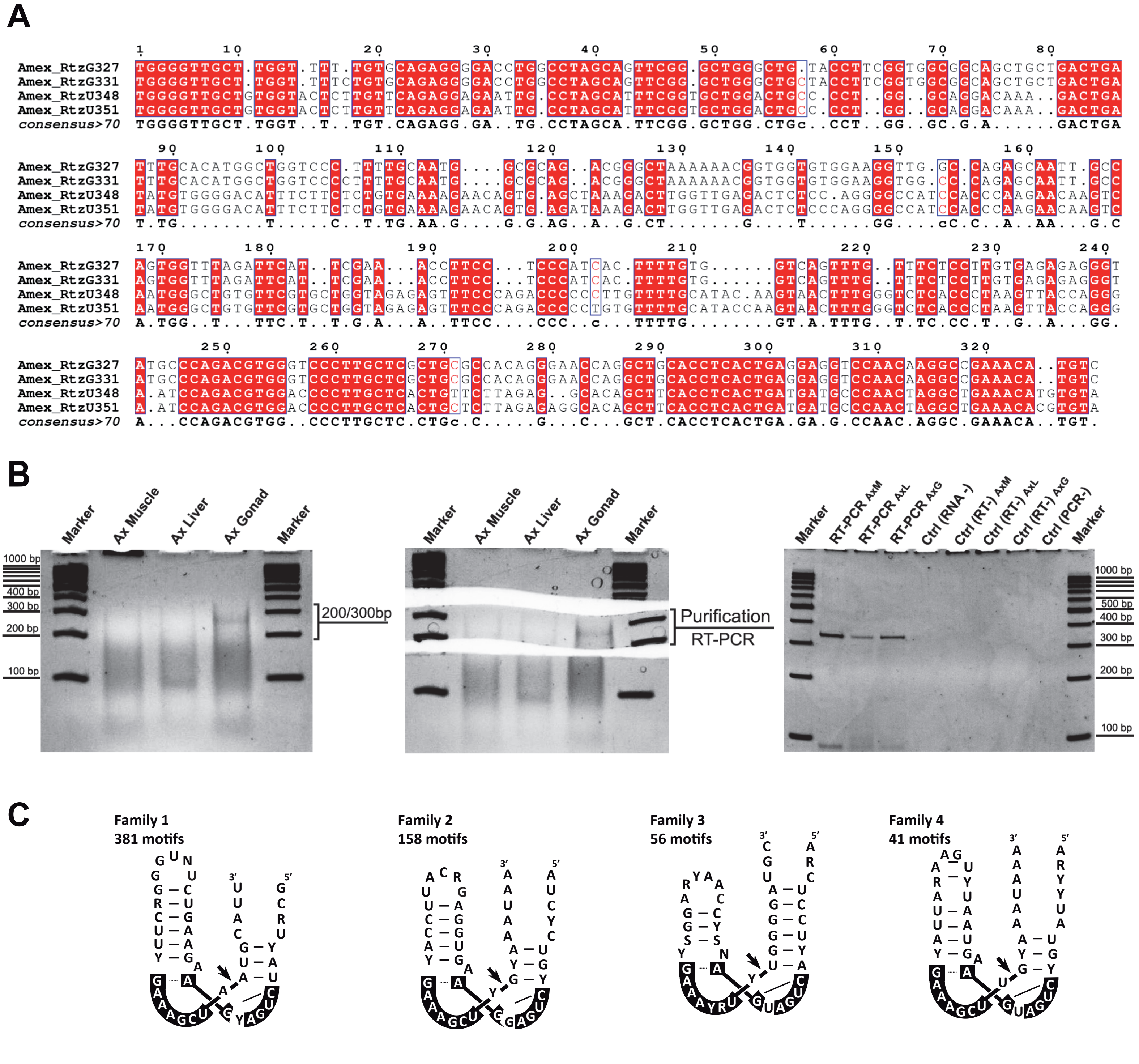

Supplementary Figure S7

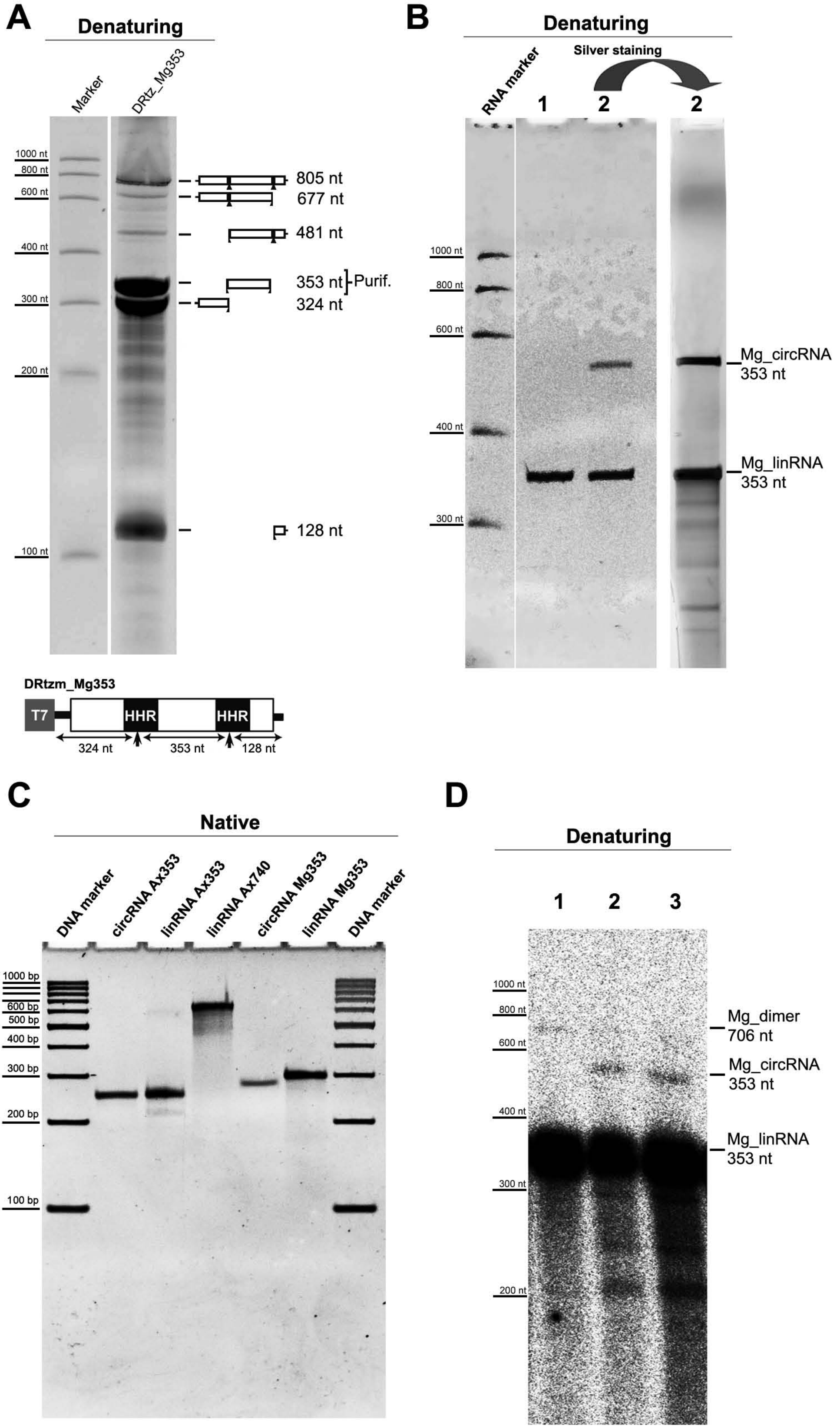

Supplementary Figure S8

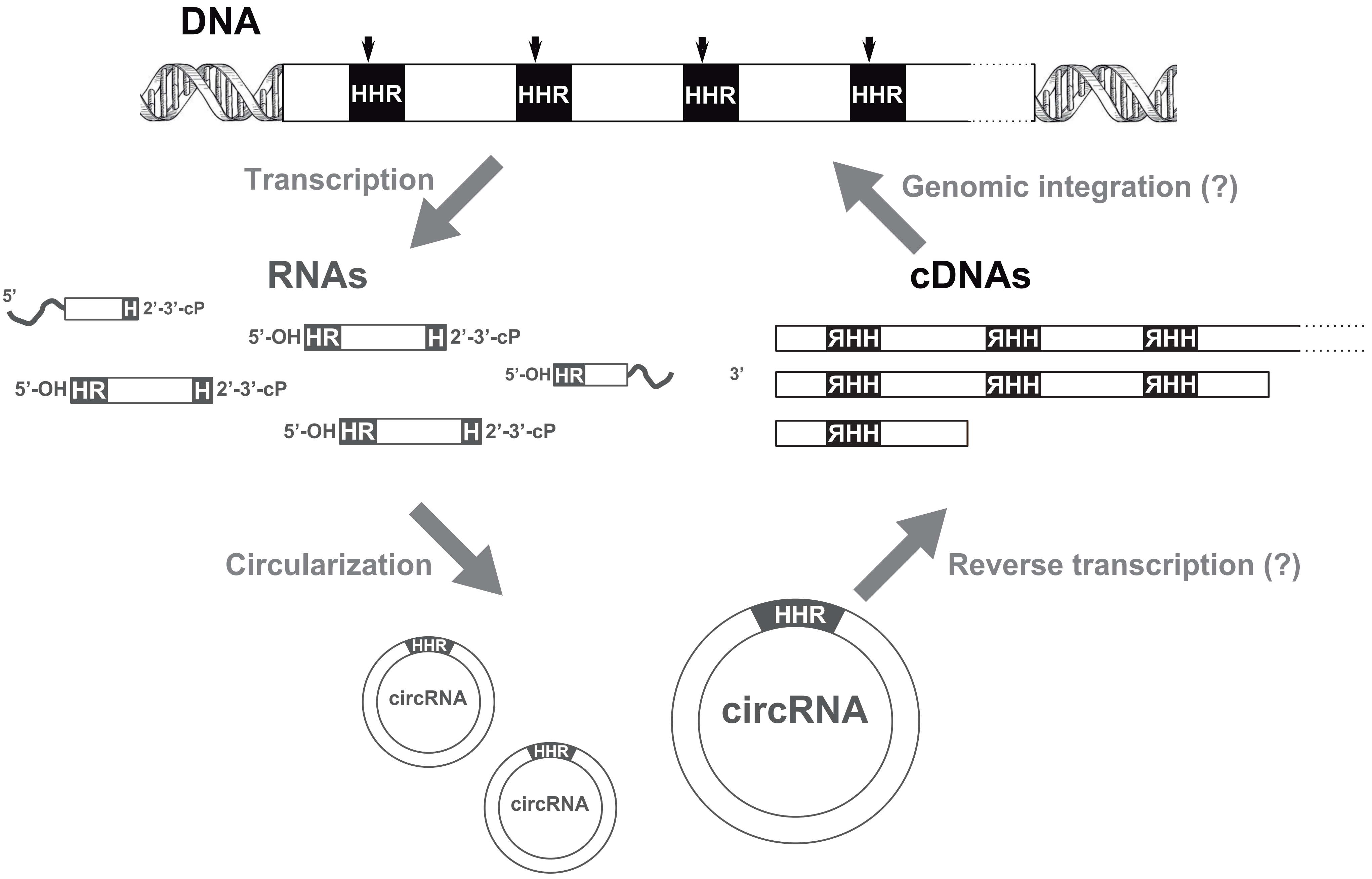

Supplementary Figure S9
